# 2348: Collaborative translational workforce development: Standardizing clinical research nursing education in good clinical practice

**DOI:** 10.1017/cts.2017.173

**Published:** 2018-05-10

**Authors:** Patricia Eckardt, Christine Kovner, Marilyn Hammer, Margaret Barton-Burke, Margaret McCabe, Elizabeth Cohn, Marie Marino, Liza Behrens

## Abstract

OBJECTIVES/SPECIFIC AIMS: The proposed pilot study seek to enhance the network of CTSAs at Rockefeller University, NYU, ISMMS, and other community members to support translational workforce development of clinical research nurses and establish a standardized nurse-specific training curriculum in GCP for use within the CTSA network, in other research centers, and in nursing school curricula. This will be coupled with a rigorous evaluation study to test the impact of the training and a comprehensive dissemination plan to make the training available to all nurses and nursing students via modern e-learning method. Aim 1. To create an integrated network of local CTSAs and community partners to develop, validate, and refine a pilot e-learning GCP educational and training program and content and outcomes dissemination plan. It is vital to integrate the efforts of CTSA leaders, community partners, and nursing educators to develop a pilot e-learning nurse workforce training curriculum and the associated evaluation measures and assessment plan. Delphi methods will be employed, coupled with rigorous assessment of face validity, content validity, and item reliability. The resulting educational training program will then be used for an e-learning educational intervention study in CTSAs, other sites, and nursing schools. Aim 2. To test the effect of the pilot GCP education and evaluation program for practicing clinical research nurses (CRNs) within the collaborating CTSAs and community partners, we will perform a randomized controlled trial using a Solomon 4 group design. For the student nurse population, we will develop a randomized control trial using a Solomon 4 group design blocked on course section. As this is a pilot study, descriptive statistics and confidence intervals around parameter estimates will be constructed. In addition, inferential statistics will be calculated on primary outcome of interest (change scores in knowledge of GCP) and measures of heterogeneity of data, patterns of missing data, and reliability of evaluative tools will be analyzed. Aim 3. To implement a dissemination plan to reach both nurses practicing the CRN specialty within CTSAs and other community settings. We will disseminate the program to other CTSAs through the CTSA network communication resources. To broaden the reach to a population of nurses and student nurses with limited prior education or training in nurse-specific GCP competencies, but who provide care to research participants in nontraditional research settings, we will craft a novel set of dissemination methods, including the CITI Program electronic platform that can be accessed by nurses and nursing students across settings. In addition, dissemination will be at nursing education meetings and in nursing journals.METHODS/STUDY POPULATION: There are several components to this pilot program. The component that includes a research strategy is the testing of the effectiveness of the training and educational interventions on GCP knowledge and efficacy. Study cohort: Recruitment of study subjects will be in coordination with 3 CTSA collaborators and community partners for 2 samples: (1) nurses who provide care to clinical research participants across a variety of settings (health care systems, research hospitals, and care provider networks) and who are already trained according to current standard in GCP, (2) nursing students from the collaborative network of the 3 CTSAs, NYU School of Nursing has agreed to pilot test the introductory student module. The methodological approach will be a random assignment control trial Solomon 4 group design for practicing CRNs within the collaborating CTSAs and community partners. For student nurse population, the methodological approach will be a randomized-control trial Solomon 4 group design blocked on course section. Survey measures of CRN GCP knowledge and efficacy will be obtained pre and post educational intervention. RESULTS/ANTICIPATED RESULTS: Aim 1. Expected outcomes are pilot e-learning nurse workforce training modules curriculum, and evaluation measures and plan appropriate for CTSAs, community sites, and nursing schools. Specifically, 14 modules (averaging 30 minutes each) for practicing CRNs, and one 45 minute module for nursing students. The significance of these findings will provide a framework for the e-learning educational intervention study. CITI Program is enthusiastic about the module development and refinement and will provide direction for consistency in formatting with current CITI Program modules, set-up of learner groups for comparison, and evaluative measures such as completion data and scoring. Aim 2. Expected outcomes are an effective pilot educational intervention for practicing nurses and students and valid and reliable evaluation tools and plan that can be generalized to the larger CRN and nursing community. Aim 3. Expected outcomes are an enhanced CTSA dissemination plan that includes non-CTSA resources and reaches non-CTSA employed nurses and nursing students. DISCUSSION/SIGNIFICANCE OF IMPACT: The expected outcomes of this pilot study are: (1) an enduring GCP education that can be continually updated and training structure for CRNs, and nurses and nursing students throughout the United States; (2) a reproducible effective standardized basic nurse-specific GCP curriculum for dissemination; (3) assessment tools to evaluate programmatic success, nurse and nursing student knowledge and efficacy on nurse-specific GCP; (4) and a CTSA dissemination plan that to reach non-CTSA nurses and nursing students. Our ultimate goal is the development of a translational workforce educated and competent in GCP at CTSA sites, at non-CTSA sites, and in nursing schools so as to improve the quality of clinical research.

